# Pseudo-labeling based adaptations of pain domain classifiers

**DOI:** 10.3389/fpain.2025.1562099

**Published:** 2025-04-23

**Authors:** Tobias B. Ricken, Sascha Gruss, Steffen Walter, Friedhelm Schwenker

**Affiliations:** ^1^ Institute of Neural Information Processing, Ulm University, Ulm, Germany; ^2^ Medical Psychology Group, University Clinic, Ulm, Germany

**Keywords:** domain adaptation, e-health, pain duration transfer, pain recognition, physiological signals, pseudo-labeling, signal segmentation

## Abstract

**Introduction:**

Each human being experiences pain differently. In addition to the highly subjective phenomenon, only limited labeled data, mostly based on short-term pain sequences recorded in a lab setting, is available. However, human beings in a clinic might suffer from long painful time periods for which even a smaller amount of data, in comparison to the short-term pain sequences, is available. The characteristics of short-term and long-term pain sequences are different with respect to the reactions of the human body. However, for an accurate pain assessment, representative data is necessary. Although pain recognition techniques, reported in the literature, perform well on short-term pain sequences. The collection of labeled long-term pain sequences is challenging and techniques for the assessment of long-term pain episodes are still rare. To create accurate pain assessment systems for the long-term pain domain a knowledge transfer from the short-term pain domain is inevitable.

**Methods:**

In this study, we adapt classifiers for the short-term pain domain to the long-term pain domain using pseudo-labeling techniques. We analyze the short-term and long-term pain recordings of physiological signals in combination with electric and thermal pain stimulation.

**Results and conclusions:**

The results of the study show that it is beneficial to augment the training set with the pseudo labeled long-term domain samples. For the electric pain domain in combination with the early fusion approach, we improved the classification performance by 2.4% to 80.4% in comparison to the basic approach. For the thermal pain domain in combination with the early fusion approach, we improved the classification performance by 2.8% to 70.0% in comparison to the basic approach.

## 1 Introduction

People learn the meaning of pain at an early stage of their lives, usually as a result of tissue damage, but also for psychological reasons, whereby the feeling itself is a complex and subjective phenomenon (1). Craig et al. (2) reported that the experience of pain is a preservative action of the human body. Apart from the individual pain perception, there are differences in experience pain between women and men, as reported in (3). These differences originate from, for instance, different coping strategies of women and men (4) or the hormone differences, as reported in (5, 6).

Pain can be categorized into acute and chronic pain: acute pain relates to pain with a short duration, often in combination with tissue damage, chronic pain relates to lasting pain present over a longer duration (7). The pain intensity level, distribution of the perceived pain and the period of the pain experience are traits of pain (8) whereby the ability of the adaption to heat pain over longer pain periods is more prominent in women than in men (9). Moreover, as reported in (10, 11), a prime cause why people seek the advice of a doctor is the experience of pain. In most cases, a patient will tell a doctor or nurse what pain they are experiencing and where it is occurring, although not all people are able to express their pain, for example due to unconsciousness or people which have communication difficulties (12). In such cases, observable behavior traits can be used by a practitioner to assess the patient’s perceived pain intensity, for instance, facial expressions or moaning (13).

The advances of observable behavior patterns might be limited due to several factors such as the socialization to pain and the belief systems of an observer which have an impact on the pain assessment of another person (2). Craig et al. (2) reported that the relationship between the observer and the person in pain affects the pain rating. In (14), the authors outlined that a pediatrician rates the experienced pain intensity of an infant lower in comparison to the parents. An observer might also be biased which might lead to over- or underestimation of the actual pain intensity a patient is suffering (15). Assessments might also be influenced by the patient’s attractiveness and hence lead to subjective rating (16).

Alternatives to a patient’s self-report and observable behavior traits are rooted in measurable biological components, such as physiological processes, which is especially helpful for patients which are not able to communicate properly, as pointed out by Korving et al. (17). With physiological measurements, an observer is able to perceive additional information in a non-invasive manner (18). The authors identified typical physiological measures used for a patient’s pain rating which are the electrodermal activity, electromyography, the heart rate variability and the heart rate through the electrocardiogram or photoplethsymography, respiration and pupillometry. The source of the alterations in the observed measures can be the experienced pain intensity, but also, for instance, medication (19).

Regardless of the pain assessment technique, it is not possible for doctors and nurses to constantly monitor a patient’s pain. However, the accurate assessment of a patient’s pain level is very important to ensure appropriate pain management that does not harm the patient (20).

The described problems in the area of pain assessment are addressed by the investigation of automatic pain recognition (APR) systems, which generally use machine learning methods for the central tasks of pain recognition. Our long-term research goal is the development of APR systems for objective pain assessment.

Most studies on automated pain assessment focus on pain assessment in combination with short-term pain stimuli recorded in a laboratory setting. However, in a hospital setting, patients are more likely to be exposed to pain over longer periods of time (21). In (9), Hashmi et al. reported that the human body habituates over time to exposed heat pain and that adaptation to heat pain is greater in women. Hence, different body reactions are expected for short-term and long-term pain elicitations, respectively, which might be reflected in the recorded physiological signals. In (22, 23), lower detection rates are achieved for segments at the end of a long-term (tonic) pain sequence in comparison to the segments in the beginning when short-term pain models are evaluated on tonic samples. Hence, an information difference between the starting and ending segments exist. Based on the reported outcomes, less information regarding the perceived pain intensity is available at the end of a tonic sequence. In addition, it is difficult to capture and accurately label tonic pain records during a patient’s hospitalization.

In this study, we address the described challenges by applying unsupervised domain matching from short-term (phasic) to long-term (tonic) pain stimuli in combination with a variety of pseudo-labeling approaches. The matching of two domains is performed by the transfer of knowledge from one (source) domain to another (target) domain (24, 25) (see Section 4.1). To this end, we apply domain knowledge from the phasic pain stimuli to pseudo-labeling tonic domain patterns by iteratively updating the training data set for our pseudo-labeling model. Our aim is to transfer phasic (source domain) pain models to the tonic pain domain (target domain) in which limited data is available. With the pseudo-labeling approaches, we aim to overcome the challenges of habituation and adaptation to pain over time with respect to pain assessment and make unlabeled tonic pain stimuli accessible to an APR system. A lot of domain adaptation approaches, which apply pseudo-labeling, focus on deep learning techniques, for instance (26–28). However, with limited data in the target domain (see Section 3) and a possible information loss within the tonic pain sequence (see above), deep learning approaches might lead to lower performances in comparison to other techniques.

Our main contributions of this paper are as follows:

1. We address the task of pain duration adaptation of pain classifiers by applying pseudo-labeling and unsupervised domain adaptation.
2. We design selection criterion for tonic pain segments to perform curriculum labeling (29) to create a pseudo-labeling model for this adaptation task.
3. We compare the performance of the evaluated approaches with the results obtained with baseline techniques.

The remaining part of this study is structured as follows. In Section 2, we summarize recent studies in the area of APR systems. In Section 3, we describe the pain database used for our study, the preprocessing steps and the feature extraction. We provide a brief formalization of the term domain adaptation and summarize the applied methods in Section 4, followed by the description of our experimental settings in Section 5. In Section 6, we present our obtained results. We discuss the outcomes in Section 7 and close this study with our conclusions and a perspective on future works in Section 8.

## 2 Related work

In this section we summarize recent outcomes in the field of pain recognition, followed by an overview of pseudo-labeling techniques.

### 2.1 Pain recognition

The field of APR systems gained a lot of interest, as it can be observed by the variety of publications with respect to evaluated pain assessment techniques, for instance, in (30–44), among others.

Semwal et al. (45) proposed a pain classification framework in which spatial and temporal data from video streams and sound is incorporated. Besides facial expressions and sound, body movements are used to assess the pain intensity. A model for each modality is created and the final outcome is based on the decision fusion.

Pouromran et al. (46), analyzed the task of continuous pain intensity level estimation on the BioVid Heat Pain Database (47). In their study, the aim was to find the best machine learning algorithm among a variety of evaluated approaches, the best signal and the best features for various pain assessment tasks. For their analysis, they extracted 22 hand-crafted time-series features which were proposed by Lubba et al. (48). The best results were obtained with the electrodermal activity (EDA) signal in combination with the Support Vector Regression algorithm. In addition, they identified the 3 most important features, specific to the EDA signal, and showed that a model trained on a reduced feature set (three statistical descriptors) achieves similar results in comparison the the model created with all 22 features. In (49), Gouverneur et al. analyzed different feature extraction techniques, specific to the EDA signal. To this end, different feature learning approaches were evaluated as well as hand-crafted features on two different pain databases. Besides standard hand-crafted features, additional EDA-specific features were extracted. To make the feature extraction techniques comparable, they presented the obtained feature vectors, specific to each approach, to a classifier which was created with the Random Forest (50) algorithm. Gouverneur et al. concluded, that simple feature extraction approaches are able to compete with complex feature learning approaches. In (51), Lu et al. proposed a deep learning architecture called PainAttnNet to learn dependencies over time within the physiological signals. The evaluation was performed on the BioVid Heat Pain Database. They obtained a state-of-the-art mean accuracy value of 85.56% with the EDA signal for the binary classification task of no pain vs. the highest pain intensity level.

Jiang et al. (52) proposed a neural network architecture which includes a block for dynamic feature attention and a fusion approach in which personalized features and classic hand-crafted features are combined. The personalization was perform by including a persons pain sensitivity. Feature extraction was applied on different sizes of sliding windows over the physiological signals. They evaluated their method on the BioVid Heat Pain Database and reported a mean accuracy value of 84.58% for the channel fusion of ECG and EDA in combination with the classification task of no pain vs. the highest pain intensity level.

Bellmann et al. (21) simulated long-term pain sequences based on randomly stacked short-term pain stimuli to analyze a more realistic pain assessment scenario. They evaluated their approach on the BioVid Heat Pain Database. In a clinical scenario, patients do not suffer from short-term pain, but from long-term or continuous pain. With their setting, the aim was to provide an upper bound, with respect to the detection of pain, in combination with long-term pain sequences. Wally et al. (53) reported initial results on the transfer learning task of phasic to tonic pain events in the electric pain domain. They designed a neural network architecture for the phasic electric domain and evaluated the created model on the unsegmented tonic electric pain events. In a previous study (22), we performed a basic pain duration knowledge transfer task in combination with distance-based approaches for the classification task of no pain vs. the highest pain intensity level, whereby we analyzed the electric and thermal pain domain, separately. The evaluation was performed on the E**x**perimentally **I**nduced **T**hermal and **E**lectrical (X-ITE) Pain database (54). To this end, each model was trained on the phasic pain domain data, whereby the model was evaluated on an individual segment, specific to a tonic sample. The selection of this segment was based on distance measures between all segments of a tonic sample and class-specific prototypes of the phasic pain domain. The segment with the lowest distance to a class-specific prototype was then presented to the model. The predicted label was used as the final label for the corresponding tonic sample.

For additional information on pain recognition, we kindly refer the reader to the following publications (18, 55) and (56).

### 2.2 Pseudo-labeling

For many of today’s data applications, only a limited amount of labeled observations are available, but huge amounts of unlabeled data points are accessible, whereby the data annotation process is cost-intensive (57). With the technique of pseudo-labeling, a semi-supervised learning approach (29), the aim is to automatically annotate the unlabeled data points. A basic approach was proposed in (58), in which the authors trained a model solely on the available labeled data points followed by predicting the class labels for the unlabeled sample. These predictions were then used as the true labels. For instance, pseudo-labeling is applied in image classification (59, 60) and face recognition (61). In (62), a similarity-based pseudo-labeling approach is proposed for the image classification in the medical domain.

Over the last decade, various pseudo-labeling approaches in combination with domain adaptation, were proposed. In domain adaptation, a model is created in a so called source domain with the objective to obtain a good performance in a so called target domain whereby for the latter no labeled data is available (see Section 4.1). For instance, based on subspace mapping and label confidence in combination with sample selection for the training process (63–66), or by analyzing the relationship between samples (67), or for semantic segmentation in combination with uncertainty (68). Saito et al. (69) proposed a pseudo-labeling approach in which a data point has to fulfill two conditions to be considered as a training sample. In their framework, two classifiers had to agree on a label for a data point and the predicted class probability (confidence with respect to the predicted label) of one of these classifiers had to be above a predefined threshold. Further, the pseudo-labeling approach of Choi et al. (26) uses curriculum labeling in combination with artificial neural networks, specific to the task of domain adaptation.

As previous studies show, for instance (64), the access to labeled target domain data is beneficial for a domain adaptation task. A broad overview of pseudo-labeling based domain adaptation approaches are provided by Li et al. (70).

## 3 Data set

In the following, we describe the pain database used in our study and the steps required to process the collected sensory data - including the extraction of the relevant features. We conclude this section with a description of the extracted feature descriptors and the total size of the databases for each pain level and stimulus type (thermal/electrical stimulation).

### 3.1 X-ITE pain database

The E**x**perimentally **I**nduced **T**hermal and **E**lectrical (X-ITE) Pain Database (54) consists of data from 67 female and 67 male subjects. All participants had no health issues at the time of the data recordings. The data was collected during experimental pain elicitation^1^ at the Ulm University.

During the experimentally induced pain, audio and video data as well as physiological signals were recorded. The video data includes recordings of facial expressions from different angles, the subject’s whole body and thermal imaging. The physiological signals are composed of the electrocardiogram (ECG), electrodermal activity (EDA) and the electromyogram (EMG). Specific for the X-ITE Pain Database is that EMG signals are collected from 3 muscles: musculus trapezius (TRA), musclus corrugator supercilii (COR) and musculus zygomaticus major (ZYG).

Two different pain stimulus types, namely electric and thermal, were applied separately to the participants of the study. Besides the stimulus type, short-term (phasic) and long-term (tonic) pain stimulation is analyzed separately, all in all four different stimulation scenarios are considered. The stimulation was performed in combination with different stimulus intensity levels.

A subject was stimulated with all available pain stimulus levels in a randomized order. A phasic pain stimulation in combination with thermal and electric pain was held for 4 and 5 s, respectively. A tonic pain elicitation always had a length of 60 s. Each subject was stimulated with each phasic pain level 30 times whereas each tonic pain level was applied only once. Each painful stimulus was followed by a no pain sequence, called baseline. The length of the phasic baselines varied between 8 and 12 s, and were randomly selected after each elicitation. The no pain sequences which followed the tonic stimuli, also called tonic baselines, had always a length of 300 s.

The data of a participant was collected in one session. For each physiological signal, the temporal resolution was set to 1000 Hz. Note that in this study, we focus on the physiological signals, i.e. the ECG, EDA and EMG signals.

### 3.2 Feature extraction

For the preprocessing and feature extraction, we followed our previous works (22, 23). Many of the extracted features are widely used in the literature, for instance in (31) based on the X-ITE Pain Database and in (30) based on the SenseEmotion (71) Database. The main steps of our feature extraction process can be briefly summarized as follows:

Based on the time window analysis in (72), the temporal windows, specific to the thermal pain elicitation, are extracted with a shift of 3 s. The time windows, specific to the electric pain elicitation, are shifted by 1 s. The time window length of each phasic stimulus is fixed to 4 s. The tonic electric and thermal time windows have a length of 57 and 59 s, respectively. As in (22, 23), each tonic stimulus is split into segments with the same window length as the phasic stimuli, whereby we ignore the last segment of each tonic stimulus due to a reduced window size of less than 4 s. Hence, a tonic stimulus is represented by 14 sequential time windows. Each signal, specific to an extracted time window, was filtered by a 3rd-order Butterworth bandpass filter, except for the EDA signal which does not show a periodic behavior. From each EMG signal, we removed the frequencies below 20 Hz and above 250 Hz. From the ECG signal, the frequencies below 0.1 Hz and above 25 Hz were removed.

From each time window, 412 statistical descriptors were extracted. From each EMG sensor signal (COR, TRA, ZYG), we extracted 82 features. From the EDA signal, we extracted 79 features. From the ECG signal, we extracted 87 features. Moreover, the EFU [early fusion, also called feature fusion in (31)] signal represents the combined feature vector of all single modalities (concatenation of the features, extracted from all modalities). In the sequel, we refer to the early fusion of the COR, TRA and ZYG signals by the EMG signal (concatenation of the features, extracted from the listed modalities).

Note that in this study, we focus on the classification task of no pain vs. the highest pain intensity level (pain tolerance level), specific to the thermal and electric pain domain, in combination with the pain duration transfer learning task. We present the amount of class-specific samples, no pain and pain in combination with each pain domain and duration type, in Table 1. For more information about the extracted time windows and the computed statistical descriptors, we kindly refer the reader to our previous works (23, 72).

**Table 1:** Samples per domain and class.

Electric domain				Thermal domain			
PB	PP	TB	TP	PB	PP	TB	TP
3,720	3,719	123	123	3,727	3,716	121	124

PB, phasic baseline; PP, phasic pain; TB, tonic baseline; TP, tonic pain, whereby baseline refers to a no pain sequence which followed a lowest pain intensity level in the corresponding domains.

## 4 Methods

In this section, we formalize the term domain adaptation and describe the general technique of pseudo-labeling. We then introduce the evaluated pseudo-labeling approaches which are applied to assign pseudo labels to the tonic segments.

### 4.1 Domain adaptation

Following (24, 25), a domain D is defined by a d-dimensional feature space X and a classifier f:X→Y, whereby Y denotes the c-dimensional label space. Let $D=\{(x_{1},y_{1}),\ldots ,(x_{n},y_{n})\}$ be a data set with $x_{i}\in X$, $y_{i}\in Y$. In a classification task, the aim is to create a classifier f based on D which leads to a good classification performance on unseen data points whereby it is assumed, that the unseen samples are drawn from the same distribution as the training samples. In a transfer learning task, two domains have to be considered which are defined as the source domain $D_{S}=(X_{S},f_{S})$ and the target domain $D_{T}=(X_{T},f_{T})$, respectively. The aim is to create a classifier in combination with the source domain data which will lead to a good classification performance in the target domain. In the literature, this scenario, in combination with the absence of target domain labels, is called unsupervised domain adaptation (UDA) (64). In the sequel, $X_{S}\in R^{n_{S}\times d}$ denotes the source domain data matrix with $n_{S}$ samples and d features. With $y_{S}$, we denote the corresponding label vector. With $X_{T}\in R^{n_{T}\times d}$, we denote the target domain data matrix, which consists of $n_{T}$ observations. The feature dimension is, again, denoted as d.

### 4.2 Pseudo-labeling based on structured prediction in UDA

In (64), Wang and Breckon proposed a pseudo-labeling approach, specific to UDA, in which they combine structured prediction and the selection of pseudo labeled observations in an iterative process. The aim is to align both domains in a dimensional reduced subspace which is learned in an iterative way by selecting pseudo labeled target domain samples for which a high confidence, with respect to the assigned label, are obtained. The approach has two tunable parameters, namely $d_{1}$ and $d_{2}$, which both represents the dimensionality of a subspace at different steps within the approach. In the sequel, we refer to this approach by the term SP approach.

### 4.3 Segmentation-based tonic pain sample pseudo-labeling

Cascante-Bonilla et al. (29) proposed an iterative curriculum labeling (CL) algorithm in which pseudo labeled samples are selected for the next training iteration when a class-specific score is above the defined confidence value. The confidence value is adapted in each iteration, and is based on the r-th percentile score, computed over the maximum class probability values of the unlabeled data set. In each iteration, r is reduced by a defined step size. Hence, the training set is able to change after each iteration. The model is always created with the labeled and currently pseudo labeled data. The algorithm terminates when all unlabeled samples are added to the training set.

In this work, we modify the approach of Cascante-Bonilla et al. to our segmentation-based problem for the domain adaptation task of phasic to tonic pain events. With this modified approach, specific for the pain domain, the idea is to overcome the effect of habituation and adaptation to pain over time, as discussed in (9, 73, 74), which might lead to false classification of segments later in time, as presented in (22, 23). Moreover, the body reactions to pain over time are reflected differently in the physiological signals. Hence, the segments over time provide different information regarding the pain intensity. Moreover, the informative content of a physiological signal is different over various segments, e.g. ECG signal behaves different in comparison to the TRA signal, pain information in the EDA signal is delayed. In our approach, we do not apply the r-th percentile score. Instead, we select all sample-specific segments, when k segments of a tonic event fulfill certain criterion, with respect to a specific class label. More precisely, we define four different criterion in combination with an iterative evaluation.

Let S be a set of segments, specific to a tonic sample, which is contained in $X_{T}$. Let s∈S be a segment, which is used as an individual data point in the training phase. Let $c_{l}\in [0,1]$ be the defined minimum confidence level. Let T be the maximum amount of iterations, performed by the algorithm. The current iteration is denoted by t. The algorithm terminates when T iterations are evaluated.

A set S is only considered as training data in the upcoming iteration, if the following criterion are fulfilled:

1. k segments of S have an averaged class-specific score above $c_{l}$, for a specific class $y_{j}$,
2. u segments of S have a class-specific score for $y_{j}$ above the chance level,
3. Each of these k segments of S, as determined above, has a confidence level above $c_{l}$ for the same class $y_{j}$
4. The absolute differences between the class-specific scores of consecutive segments of a tonic event are below q on average.

In addition, we only use p% of the source domain samples in each iteration. With these requirements, we aim to create a model that is able to perform an adequate pain assessment on all segments of a tonic observation. In addition, if the requirements are fulfilled for the pain class, we do not evaluate the requirements for the baseline class since the pain detection is more challenging, as described above. Further, if only the first two conditions are fulfilled for the pain class, but the third and fourth conditions are not, we do not evaluate the conditions for the no pain class and vice versa, for the same reason. Note that we always favor the pain class. On termination, the final model is returned, which then can be used to pseudo-labeling the tonic observation-specific segments.

An algorithmic overview is depicted in Algorithm 1. In the sequel, we will refer to the adapted curriculum labeling approach with the term ACL approach. To the best of our knowledge, no such pseudo-labeling approach in combination with signal segmentation exists in which all segments, specific to a time series signal, are selected based on a subset of these segments in combination with favoring one class (pain) over the other class (no pain).

**Algorithm 1:** ACL algorithm, modified version of the curriculum labeling algorithm (29), for the defined pain recognition task.

**Input:** k, $c_{l}$, u, q, p, T, classifier-specific settings **Output:** $C_{t}$ 1: $X_{S^{\prime }},y_{S^{\prime }}\leftarrow \text{randomly select }p\%\text{ source domain samples}$ 2: $C_{0}$ $\leftarrow \;\text{classifier trained on }(X_{S^{\prime }},y_{S^{\prime }}$) 3: t←0 4: **while** non of the termination criteria is reached **do** 5: $X_{\mathrm{new}}\leftarrow []$ {holds segment sets $S\in X_{T}$ considered as training samples for the next iteration} 6: t←t+1 7: **for** $S\in X_{T}$ **do** 8: $y_{t}\leftarrow C_{t-1}$(S) {decision vectors specific to S} 9: $S_{y_{P}}\leftarrow k$ segments highest pain class scores $y_{t}$ 10: $S_{y_{B}}\leftarrow k$ segments highest baseline class scores $y_{t}$ 11: **if** $S_{y_{P}}$ fulfills 1. and 2. criterion **then** 12: **if** $S_{y_{P}}$ fulfills 3. and 4. criterion **then** 13: $X_{\mathrm{new}}\leftarrow X_{\mathrm{new}}\cup S$ 14: **else** 15: do nothing {S is not considered} 16: **end if** 17: **else if** $S_{y_{B}}$ fulfills 1. and 2. criterion **then** 18: **if** $S_{y_{B}}$ fulfills 3. and 4. criterion **then** 19: $X_{\mathrm{new}}\leftarrow X_{\mathrm{new}}\cup S$ 20: **else** 21: do nothing {S is not considered} 22: **end if** 23: **else** 24: do nothing {S is not considered} 25: **end if** 26: **end for** 27: $X_{S^{\prime }},y_{S^{\prime }}\leftarrow \text{randomly select }p\%\text{ source domain samples}$ 28: **if** $X_{\text{new}}$ is empty **then** 29: $X_{t-1}\leftarrow X_{S^{\prime }}$, $y_{t-1}\leftarrow y_{S^{\prime }}$ {no segments selected} 30: **else** 31: $X_{t-1}\leftarrow X_{S^{\prime }}\cup X_{\mathrm{new}}$, $y_{t-1}\leftarrow y_{S^{\prime }}\cup C_{t-1}(X_{\mathrm{new}})$ 32: **end if** 33: $C_{t}$ $\leftarrow \text{classifier trained on }X_{t-1},y_{t-1}$ {trained from scratch} 34: **end while** 35: 36: **return** $C_{t}$

## 5 Experimental settings

In this section, we describe our experimental setup and the parameter settings for the pseudo-labeling approaches.

In this study, we perform the classification task of no pain vs. the highest pain intensity level (in the sequel: no pain vs. pain) in combination with the classifier adaptation from phasic to tonic pain events, whereby we focus on the physiological signals. Note that each segment (see Section 3) in the training set is used as an individual sample. To this end, we evaluate three different pseudo-labeling approaches, i.e. naive pseudo-labeling (NAP) approach [similar to (58), described in Section 4], the SP approach (see Section 4.2), the ACL approach (see Section 4.3), and analyze the performances in combination with each uni-modal signal and the multi-modal signals (see Section 3), specific to the electric and thermal domain. An overview of the evaluated approaches is presented in Table 2.

**Table 2:** Summary of the evaluated approaches.

Abbreviation	Approach
Ref.	Reference Value: No pain vs. the highest pain level in the tonic pain domain. No segmentation is applied. A tonic pain domain model is trained with the true labels of the tonic samples. The model is evaluated on tonic domain samples.
NAS	No Adaptation (segments): Presenting segments of the tonic stimulus to the phasic pain domain classifier whereby the final label is obtained by a majority vote.
UB	Upper Bound: Perfect pseudo labels. The training set contains the labeled phasic stimuli and labeled tonic stimuli segments. The evaluation is performed on tonic segments (majority vote).
NAP	Naive Pseudo-Labeling: A phasic pain domain model is applied to the tonic pain domain segments to assign pseudo labels. A model is trained on the phasic domain samples and pseudo labeled tonic segments. The evaluation is perform on tonic segments (majority vote).
SP	Structured Prediction proposed in (64): Iterative approach to alignment the source and target domain in a subspace. A model is then trained on the aligned domain data (phasic samples and tonic segments). The model is evaluated on tonic domain segments (majority vote).
ACL	Adapted Curriculum Labeling: The modified CL approach which we introduce in Section 4.3. Iterative training set updates with confident pseudo labeled tonic domain segments according to our defined requirements. A final model is then trained on the phasic pain domain samples and the tonic pain domain segments. The model is evaluated on tonic domain segments (majority vote).

The performance of each approach is measured by the accuracy, due to the almost equal amount of samples for the pain and no pain classes (see Table 1). The applied evaluation protocol is the leave-one-subject-out cross-validation (LOSO-CV) approach. In each iteration, we use the tonic events of the left out subject as the test set. The final performance score is given by the averaged accuracy over the LOSO-CV. Specific to one LOSO-CV iteration, a classifier is created with the Random Forest (RF) algorithm (50), as in (22, 23, 72). A comparison of classifier types in (35, 49) showed that RF models in combination with hand-crafted features can lead to competitive results in comparison to results obtained with deep learning approaches and other types of classifiers. Each RF uses 100 Decision Trees (75) (DTs), whereby the maximum depth is restricted to 10. The Gini index is used to rate the split quality.

In the evaluation of the reference approach, the training set of one LOSO-CV iteration is constituted of tonic domain samples of n−1 subjects only. In the NAS approach, the training set of one LOSO-CV iteration contains only phasic domain samples of n−1 subjects.

In the evaluation of the approaches UB, NAP, SP and ACL, the training set of one LOSO-CV iteration is constituted of phasic domain samples and the pseudo labeled tonic domain segments of n−1 subjects. With this set, a classifier is trained from scratch and tested on the segments of the left out subject. For each tonic sample in the test set, 14 decision vectors, one for each segment, are obtained. We compute the class-specific average score over the decision vectors and assign the class label with the highest score to the tonic event.

For the NAP approach, a phasic domain model is used to assign pseudo labels to the segments in the training set. The optimal subspace dimensions in the SP approach are estimated by conducting a grid search over $d_{1}\in \{50,60,70\}$ and $d_{2}\in \{20,30,40\}$ in combination with 5 iterations. Specific to the ACL approach, the values $c_{l}\in \{0.65,0.70,0.75,0.80,0.85,0.90\}$ are evaluated in combination with 5,10,20 and 30 iterations. We set the minimum number of segments k to 7, the amount of used source domain data p is set to 80%, u=10 and q=0.2. Note that we construct each RF classifier in the ACL algorithm with the same settings as described above.

An upper bound (UB) is evaluated in which we simulate perfect pseudo-labeling by using the true labels.

As the reference values (Ref.), we provide the obtained results from the no pain vs. pain task in the tonic domain whereby we use the unsegmented signal. Moreover, we create baseline results in which a model is solely trained on phasic data, i.e. the segmentation-based naive (NAS) approach (model evaluated on segmented tonic events, label assigned as described above).

**Figure 1:**
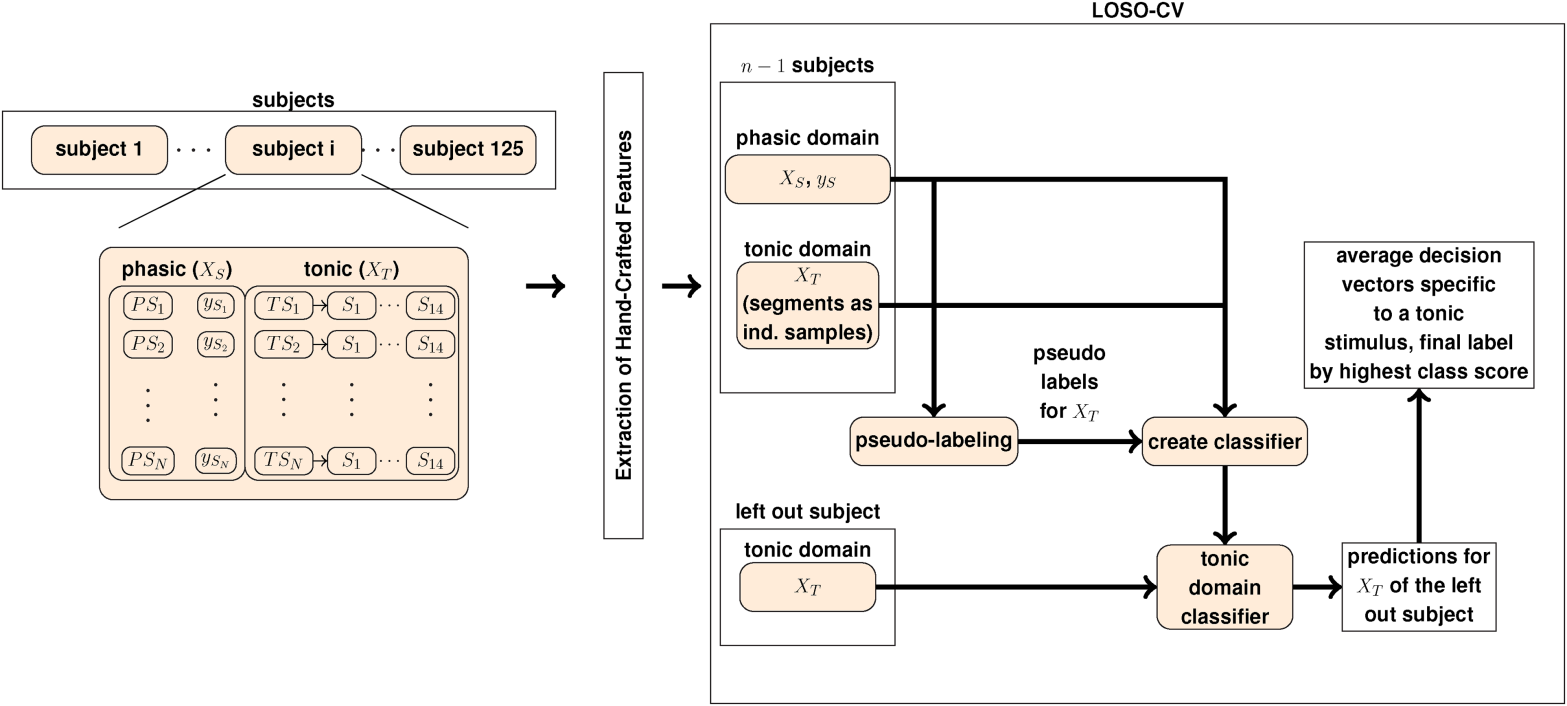
The pseudo-labeling experiment pipeline to evaluate each pseudo-labeling approach. $PS_{i}$ denotes a phasic event with the corresponding label $y_{S_{i}}$. $TS_{I}$ denotes a tonic observation whereby the corresponding segments are denoted by $S_{J}\in \{1,\ldots ,14\}$. $X_{S}$ and $y_{S}$ is the set of phasic (source) domain observations and the corresponding label vector, respectively. The set of tonic domain segments is denoted by $X_{T}$.

The standardization of the data is implemented by applying the z-score (zero-mean, unit-variance). More precisely, for each participant the phasic baseline and phasic pain tolerance stimuli, specific to the electric and thermal domain, are selected. Then, the z-score is computed over the combined participant-specific phasic electric and phasic thermal domain datasets, respectively. The same standardization is applied to the tonic domain, in combination with the reference task. The standardization approach is different to (22, 23, 72) and leads to distinct results for the reference approach in comparison to the literature. For the segments in the training set, we apply the same approach as for the phasic pain events. Each tonic sample in the test set is standardized by computing the z-score over the sample-specific segments. For our experiments, we use the Python programming language in combination with the Python data stack (76–80). An overview of the experiment pipeline is depicted in Figure 1.

## 6 Results

In this section, we present the obtained results for the classifier adaptation from phasic to tonic pain based on the described pseudo-labeling approaches (see Section 4), whereby we measure the performance on the classification accuracy of no pain vs. pain.

First, we present the achieved results in the electric domain, followed by the obtained results in the thermal domain. We close each domain-specific section with a comparison of the evaluated approaches.

Note that for the pseudo-labeling approaches, the training set of one LOSO-CV iteration is constituted of phasic domain samples and the pseudo labeled tonic domain segments of n−1 subjects. For the UB approach, the true labels are used. A model is then trained on the created training set and evaluated on the segments, specific to the tonic samples, of the left out subject (see also Section 5).

### 6.1 Electric pain stimulation

#### 6.1.1 SP

The best performing $d_{1}$ and $d_{2}$ settings, specific to each signal, are presented in Table 3. We also evaluated the best performing settings in combination with 10 and 20 iterations, but did not observe any improvements.

**Table 3:** Electric domain SP approach: the obtained signal-specific accuracy values (Acc.) of the best parameter settings, in combination with 5 iterations.

Settings and Acc.	Signal						
Parameter	COR	TRA	ZYG	ECG	EDA	EMG	EFU
$d_{1}$	70	50	60	50	50	50	60
$d_{2}$	20	40	30	40	20	30	40
Acc.	59.6	80.8	60.8	54.4	56.0	76.8	72.6

The results are given in %.

The best performing modalities are the TRA (80.8%), EMG (76.8%) and EFU (72.6%) signals. The lowest performance is observed for the ECG signal (54.4%).

#### 6.1.2 ACL

We present a detailed overview of the achieved results, specific to each signal, in Table 4.

**Table 4:** Electric domain ACL approach: the obtained results for the evaluated *c_l_* values in combination with 5, 10, 20 and 30 iterations, specific to each signal.

Parameter		Signal						
	$c_{l}$	COR	TRA	ZYG	ECG	EDA	EMG	EFU
5 Iterations	0.65	58.8	72.4	55.6	51.2	50.0	78.4	78.4
	0.70	58.0	71.6	57.2	50.8	51.6	76.0	78.4
	0.75	58.4	73.2	54.0	55.2	50.4	75.6	77.6
	0.80	57.6	71.6	55.6	54.0	53.2	74.0	80.0
	0.85	57.6	72.0	55.6	52.4	58.8	76.0	77.2
	0.90	56.4	70.8	54.0	56.8	58.0	76.0	77.6
10 Iterations	0.65	61.2	72.8	54.4	50.0	49.6	76.0	79.2
	0.70	58.8	73.2	56.0	49.2	49.2	75.2	78.0
	0.75	58.0	73.6	58.4	54.4	49.6	77.2	78.0
	0.80	58.0	72.8	54.8	55.6	52.0	75.6	80.4
	0.85	59.6	72.0	54.4	52.4	60.0	74.8	78.0
	0.90	58.0	70.4	54.8	53.6	60.0	73.6	76.4
20 Iterations	0.65	59.4	73.2	56.8	50.0	49.2	76.8	76.4
	0.70	57.2	73.2	55.2	50.0	49.6	76.4	76.8
	0.75	58.8	70.8	57.6	51.6	50.8	73.6	79.6
	0.80	60.0	73.6	54.8	53.6	51.8	75.6	78.8
	0.85	57.6	73.2	56.8	52.8	62.8	74.8	79.6
	0.90	58.0	71.2	52.4	54.0	57.6	73.6	77.2
30 Iterations	0.65	61.6	74.4	54.8	48.4	50.4	76.8	78.8
	0.70	58.8	73.6	55.6	50.8	50.0	75.6	79.2
	0.75	58.0	72.8	57.2	52.0	50.8	75.6	78.0
	0.80	58.0	73.2	57.2	56.8	50.8	75.2	79.6
	0.85	57.6	73.6	54.4	54.4	57.6	74.0	78.0
	0.90	57.2	72.4	55.2	54.0	60.4	72.8	77.2

The results are given in %. The highest outcomes are depicted in bold.

The best performing modalities are the EFU (80.4%) and the EMG (78.4%) signals over 10 and 5 iterations, respectively. The lowest mean accuracy value is obtained for the ECG signal (56.8%) in combination with 5 iterations and $c_{l}=0.9$, as well as for 30 iterations and $c_{l}=0.8$. In most cases, a $c_{l}$ value of 0.9 (high confidence with respect to the label of a segment) did not improve the outcomes. Especially for the EMG signal, higher $c_{l}$ values led to lower outcomes. An increase of the number of iterations did not necessarily improve the performance.

#### 6.1.3 Comparison

In Table 5, we present the highest obtained accuracy rates in combination with the pseudo-labeling approaches, including the reference values and baseline results, specific to the electric domain.

**Table 5:** Electric domain: summary of all obtained results, specific to each signal and approach (APPR), given in %.

APPR	Signal						
	COR	TRA	ZYG	ECG	EDA	EMG	EFU
Ref.	68.4	82.8	67.2	79.2	82.0	85.2	88.4
NAS	56.8	70.4	56.4	50.8	$\underline{67.2}$	77.2	72.8
UB	60.0	70.4	57.2	54.0	59.2	73.6	76.4
NAP	58.8	74.0	54.8	54.4	56.8	76.8	78.0
SP	59.6	$\underline{80.8}$	$\underline{60.8}$	54.4	56.0	76.8	72.6
ACL	$\underline{61.6}$	73.6	58.4	$\underline{56.8}$	62.8	$\underline{78.4}$	$\underline{80.4}$

A bold value denotes the highest accuracy value among the evaluated pseudo-labeling approaches. An underlined value denotes the highest outcome among all evaluated approaches.

For each modality the NAS and NAP approaches are outperformed by the ACL or SP approaches, except for the EDA signal. Moreover, for each signal, the UB approach is outperformed by one of the evaluated pseudo-labeling approaches. For the EMG and EDA signals, the NAS approach outperforms the UB approach.

The SP approach in combination with the TRA signal leads to a maximum of 80.8% which is close to the within domain result (Ref.: 82.8%). For the EMG signal, the highest classification performance of 78.4% is obtained with the ACL approach (1.6% above the NAP and SP approaches: 76.8%, 1.2% above the NAS approach).

For the EDA signal in combination with the NAS approach, a maximum of 67.2% is obtained which is the highest achieved outcome for the EDA modality. For the EFU signal, the ACL approach (80.4%) outperformed the NAP approach by 2.4% and the SP approach by 7.8% and leads to a higher classification performance in comparison to the UB approach (76.4%).

The highest classification performance is yielded by the SP approach in combination with the TRA signal (80.8%). The lowest accuracy value is observed for the ECG signal (ACL approach: 56.8%).

### 6.2 Thermal domain stimulation

#### 6.2.1 SP

The signal-specific best performing $d_{1}$ and $d_{2}$ settings, are presented in Table 6. We also evaluated the best performing settings in combination with 10 and 20 iterations, but we did not obtain improved results. The best performing modality is the EFU signal for which a mean accuracy value of 69.2% is obtained. The worst performing modality is the ECG signal for which we obtained a mean accuracy value of 53.6%.

**Table 6:** Thermal domain SP approach: the obtained signal-specific accuracy values (Acc.) of the best parameter settings, in combination with 5 iterations.

Settings and Acc.	Signal						
Parameter	COR	TRA	ZYG	EMG	ECG	EDA	EFU
$d_{1}$	60	70	70	60	60	50	70
$d_{2}$	40	40	30	40	30	40	20
Acc.	58.0	57.2	64.4	66.4	53.6	62.4	69.2

The results are given in %.

#### 6.2.2 ACL

We present a detailed overview of the achieved results in Table 7. For the EFU signal, an increase of the $c_{l}$ value led to higher outcomes in combination with each evaluated number of iterations. A maximum of 70.0% is obtained for $c_{l}$ set to 0.90 in combination with 5 and 20 iterations. This is also the best performing modality. The variation of the obtained outcomes are mainly based in the random source domain data selection in which more combinations are evaluated when the termination of the algorithm is extended. For the EMG signal, similar to the outcomes of the EFU signal, higher $c_{l}$ values led to improved outcomes whereby the highest accuracy value of 66.0% is obtained with $c_{l}=0.90$ in combination with 20 iterations. With the ECG signal, we obtained the lowest classification performance (54.4%).

**Table 7:** Thermal Domain ACL approach: the obtained results of the best parameter settings, in combination with 5, 10 and 20 iterations, specific to each signal.

Parameter		Signal						
	$c_{l}$	COR	TRA	ZYG	ECG	EDA	EMG	EFU
5 Iterations	0.65	55.6	52.0	62.8	51.2	56.0	60.0	66.0
	0.70	54.4	51.6	62.8	52.4	57.2	63.6	64.8
	0.75	59.2	52.8	65.6	52.0	58.8	60.0	68.8
	0.80	57.2	51.6	64.0	53.2	58.8	62.0	69.6
	0.85	56.0	54.8	64.8	53.6	60.4	62.0	69.6
	0.90	57.6	54.8	65.6	52.8	61.2	64.0	70.0
10 Iterations	0.65	56.8	52.0	62.0	51.6	55.2	59.2	64.0
	0.70	55.6	52.8	64.4	50.0	57.2	61.2	64.8
	0.75	56.4	52.8	62.0	52.8	58.8	61.6	64.8
	0.80	56.0	54.4	63.2	52.4	58.0	62.4	68.0
	0.85	59.2	51.2	62.0	50.4	61.2	63.2	67.2
	0.90	57.6	53.2	60.8	53.2	60.0	62.8	68.8
20 Iterations	0.65	55.2	50.4	62.8	52.4	57.6	60.0	63.6
	0.70	55.2	52.4	62.8	50.4	61.2	60.4	65.6
	0.75	56.8	51.6	63.6	50.0	58.4	62.0	67.2
	0.80	55.6	52.8	66.4	52.0	56.8	62.8	69.2
	0.85	58.8	53.2	63.6	53.6	59.6	62.0	69.6
	0.90	58.0	54.8	62.4	53.2	58.8	66.0	70.0
30 Iterations	0.65	56.8	50.8	60.8	53.6	56.4	58.8	63.2
	0.70	56.8	52.0	62.8	50.4	61.6	60.4	62.8
	0.75	57.2	51.6	63.6	49.6	57.6	60.8	65.6
	0.80	55.6	53.6	65.2	54.4	56.0	62.8	68.8
	0.85	58.0	53.6	64.0	51.6	58.4	62.0	67.6
	0.90	58.0	53.6	62.8	50.4	61.2	65.2	68.8

The results are given in %. A bold value denotes the highest accuracy value among the evaluated approaches.

#### 6.2.3 Comparison

In Table 8, we present the highest obtained accuracy rates in combination with the pseudo-labeling approaches, including the reference values and baseline results, specific to the thermal domain.

**Table 8:** Thermal domain: summary of all obtained results, specific to each signal and approach (APPR), given in %.

APPR	Signal						
	COR	TRA	ZYG	ECG	EDA	EMG	EFU
Ref.	62.0	56.0	57.6	64.8	79.2	61.6	79.2
NAS	53.6	50.4	60.4	53.2	58.8	62.8	66.4
UB	52.4	53.2	55.6	$\underline{56.4}$	$\underline{65.2}$	59.2	67.2
NAP	58.4	54.4	63.6	49.6	62.8	64.4	67.2
SP	58.0	$\underline{57.2}$	64.4	53.6	62.4	$\underline{66.4}$	69.2
ACL	$\underline{59.2}$	54.8	$\underline{66.4}$	54.4	61.6	66.0	$\underline{70.0}$

A bold value denotes the highest accuracy value among the evaluated pseudo-labeling approaches. An underlined value denotes the highest outcome among all evaluated approaches.

For the TRA signal in combination with the SP approach, we obtained a classification performance of 57.2%, which is 1.2% above the reference value (56.0%). For the ZYG signal, a maximum of 66.4% is obtained in combination with the ACL approach, an improvement of 10.8% in comparison to the UB approach and 8.8% above the reference value. For the EMG signal, the SP approach leads to a maximum of 66.4%, whereby the UB approach is outperformed by 7.2%. A slightly lower performance was observed for the ACL approach (66.0%).

The best performing modality is the EFU signal with a maximum of 70.0%, an improvement of 2.8% in comparison to the UB approach. The lowest performance is observed for the TRA signal (57.2%, SP approach).

## 7 Discussion

In this study, we evaluated a variety of experiments on the classifier adaptation from phasic to tonic pain domains, based on different pseudo-labeling approaches. To this end, we analyzed the task of no pain vs. the highest pain intensity level. We rated the performance of each approach by the classification accuracy of the obtained model in the tonic domain.

Our findings show that we are able to provide valuable knowledge to a classifier, based on the pseudo labeled segments. Since the overall performance improves with pseudo-labeling, a training set, constituted of phasic events and pseudo labeled segments, should be considered.

Higher accuracy values are observed in the electric domain, in comparison to the thermal domain, similar to (22, 23). Moreover, as already discussed in previous studies, for instance (31, 72), the electric elicited pain is felt instantly whereby for thermal stimulated pain, the elevation of the temperature needs time. Analogously, the electric elicited pain stops instantly when the stimulus is removed, which is different to thermal stimulation. Furthermore, no evaluated approach performs equally well in both domains and on all modalities.

Due to the differences of the z-score computation (see Section 5), with respect to the segments in the training and test sets, the adaptation task might become more challenging. Based on that, a shift between the tonic segments of these sets was implemented. However, in a clinical scenario, the training data might not be available due to privacy concerns. Therefore, the standardization has to be performed only on the patient’s data. Hence, a different approach might improve the results.

For each signal in the electric domain, we outperformed the UB approach by at least one pseudo-labeling technique. We observed a similar outcome in the thermal domain, except for the ECG and EDA signals. Hence, the good adaptation of the models to the true labeled segments might be an additional issue, whereby the inaccurate pseudo labels led to an improved generalization, with respect to unseen tonic segments.

Moreover, with a pseudo-labeling approach, the NAS approach is always outperformed, except for the EDA signal in combination with the electric domain. In Table 9, we present our highest obtained outcomes, based on the pseudo-labeling approaches, and previous reported classification performances, specific to the pain duration adaptation task. In most cases, we outperformed the previously achieved accuracy values.

**Table 9:** Our results in comparison to previous studies.

Domain	Approach	Signal						
		COR	TRA	ZYG	ECG	EDA	EMG	EFU
Electro	(22)	54.0	68.0	54.8	56.8	54.0	67.2	60.8
	(23)	–	–	–	–	–	–	69.2
	(53)	–	–	–	–	–	–	65.2
	Pseudo-labeling	61.6	80.8	60.8	56.8	62.8	78.4	80.4
Thermal	(22)	55.2	60.0	58.8	55.2	59.6	57.6	57.6
	(23)	–	–	–	–	–	–	67.6
	(53)	–	–	–	–	–	–	–
	Pseudo-labeling	59.2	57.2	66.4	54.4	62.8	66.4	70.0

Bold marked values denote the highest classification performances. In (22, 23), tonic samples are split into segments whereby in (53) the tonic samples are used without additional segmentation. In (22, 23, 53), the models are trained on phasic pain domain samples and evaluated in the tonic pain domain.

### 7.1 Electric pain stimulation

The highest performance was obtained with the TRA signal in combination with the SP approach (80.8%). The highest accuracy values in combination with the SP approach (Table 3) were obtained, with the maximum number of iterations set to 5. More iterations did not lead to improved results, which was already observed in (64) for different tasks.

**Figure 2:**
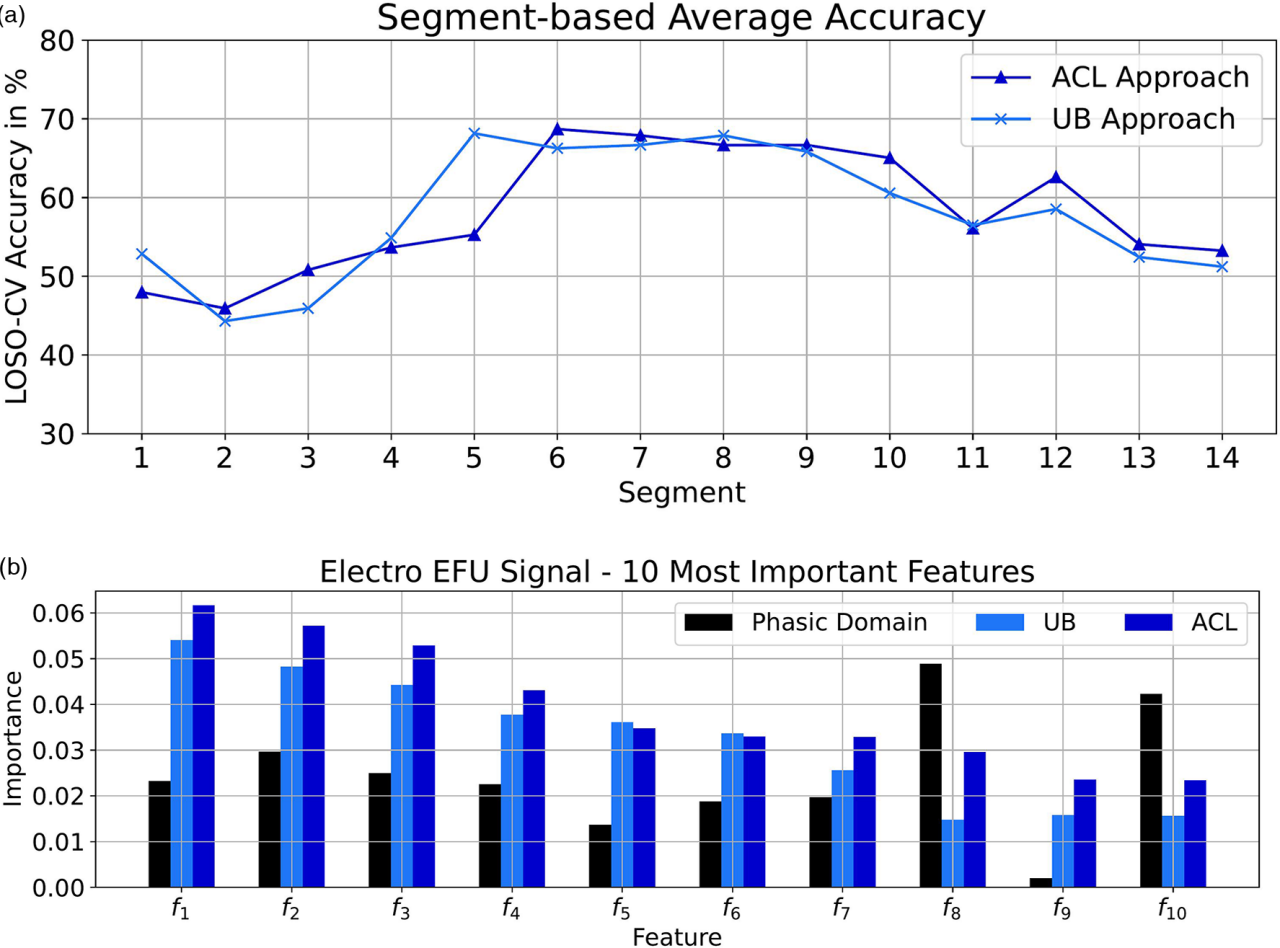
Electric domain: (**a**) Segmentation-based average accuracy EFU signal. (**b**) Ten most important features EFU signal. (**a**) EFU signal: The segment-specific accuracy values for the UB and ACL approaches in the electric domain. (**b**) The determined ten most important features, specific to the EFU signal in combination with the ACL approach, in the electric domain. A difference, among the approaches ACL and UB as well as the phasic domain, with respect to the feature importance is observable. In Table 10, we present the names of the most important EFU features for the electric pain domain in combination with the ACL approach.

We analyzed the performances of the EFU signal in combination with the UB and ACL approaches on the signal-segment level. The obtained performance values are depicted in Figure 2a. We only achieved small improvements on the ending segments of the tonic pain events (segments 10, 12, 13 and 14). However, these improvements in combination with the similar outcomes on the remaining segments, in comparison to the UB approach, lead to an increased classification performance by 4.0%, specific to the electric EFU signal (80.4%).

**Table 10:** Electric domain: EFU ACL important features.

$f_{n}$	Feature name
$f_{1}$	Trap. 2. derivative signal max
$f_{2}$	Trap. 2. derivative signal var
$f_{3}$	Trap. 2. derivative signal max to min peak value ratio
$f_{4}$	Trap. 2. derivative signal std
$f_{5}$	Trap. 2. derivative signal range
$f_{6}$	Trap. 2. derivative signal rms
$f_{7}$	Trap. 2. derivative signal split equal part mean
$f_{8}$	Scl signal max
$f_{9}$	Trap. 1. derivative signal var
$f_{10}$	Scl signal range

Moreover, we analyzed the ten most important features, with respect to the EFU signal. To determine these features, we followed the approach of Gouverneur et al. (35) for the collection process. In each LOSO-CV iteration, we gathered the importance score of each feature, specific to the ACL approach. We averaged the obtained feature importance vectors^2^ and selected the ten features with the highest scores. We applied the same process on the phasic domain models and the UB approach, to obtain the feature importance vectors. We then picked the scores from these feature importance vectors, specific to the selected ACL features. These scores are depicted in Figure 2b. For more details about the feature computation, we kindly refer the reader to the papers (23, 72).

As it can be seen, for each approach, the feature-specific importance is different. Despite the imperfect pseudo labels, we were able to create models which are shifted in direction of the tonic domain, which is observable by the changes in the scores and the improved classification performance.

Furthermore, we outperformed the basic approach (NAS) with at least one pseudo-labeling technique, except for the EDA signal (Table 5). For the EDA signal, the highest obtained outcome was 67.2% (NAS approach). We assume that a high similarity between the data of the phasic events and the segments in combination with the sample-specific standardization approach exist which leads to the promising outcome. Since the obtained results, in combination with the UB and pseudo-labeling approaches, are below the NAS approach, we conclude that the reflected similarity in the model was removed by the approaches and the z-score computation of the training set segments. Therefore, the model was not shifted in direction of the tonic events and led to lower outcomes.

### 7.2 Thermal pain stimulation

For the COR, ZYG and EMG signals, the UB approach is outperformed by the NAS approach (Table 8). The highest performance was obtained with the EFU signal in combination with the ACL approach (70.0%).

**Figure 3:**
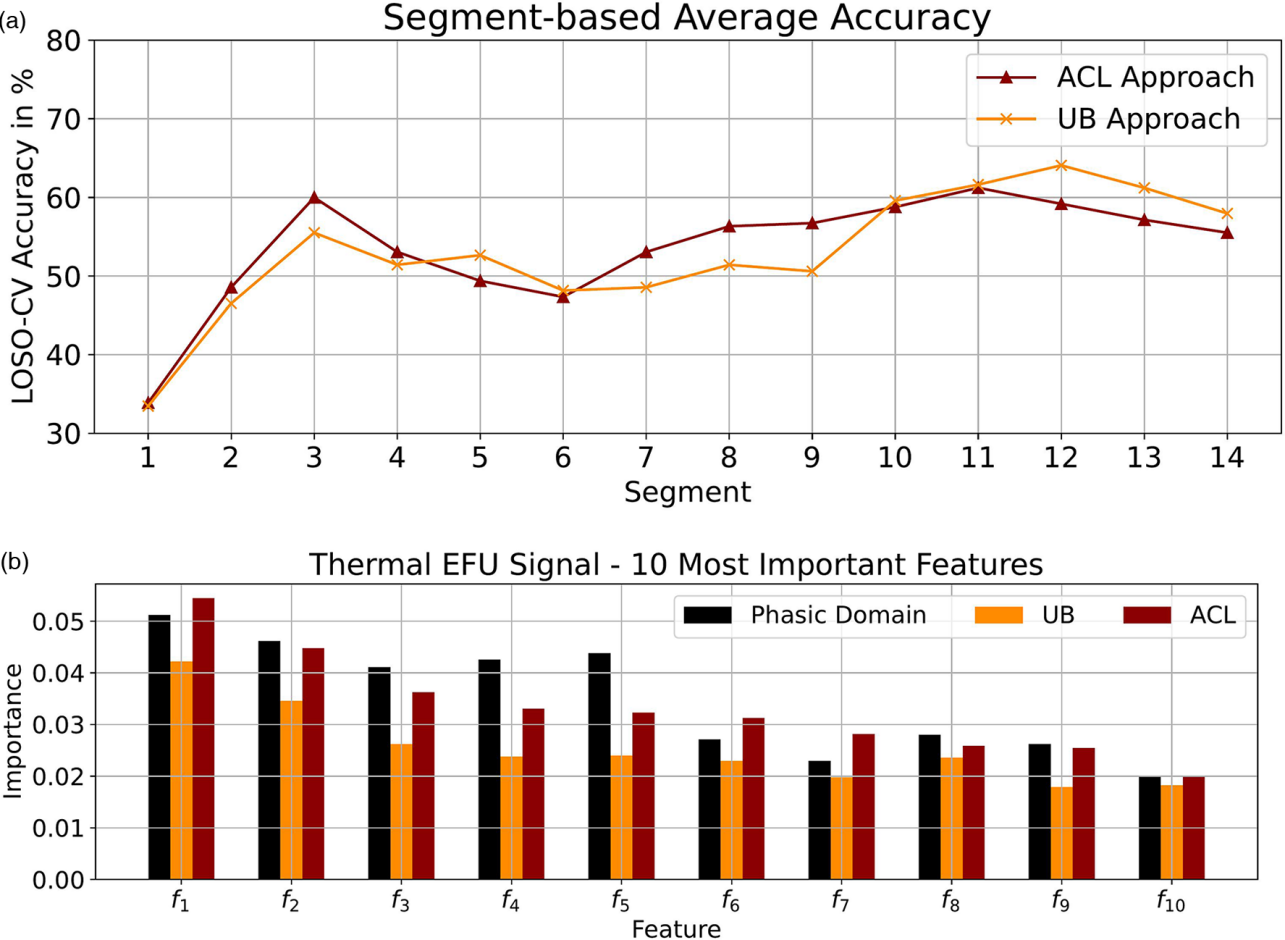
Thermal domain: (**a**) Segmentation-based average accuracy EFU signal. (**b**) Ten most important features EFU signal. (**a**) EFU signal: The segment-specific accuracy values for the UB and ACL approaches in the thermal domain. (**b**) The determined ten most important features, specific to the EFU signal in combination with the ACL approach, in the thermal domain. A difference, among the approaches ACL and UB as well as the phasic domain, with respect to the feature importance is observable. The most important features for the EFU signal in combination with the ACL approach are depicted in Table 11.

Due to the promising outcome for the EFU signal, we further investigated the performances of the UB and ACL approaches by analyzing the segmentation-based accuracy values, which are depicted in Figure 3a. On average, with the ACL approach, we obtained slightly higher accuracy values for the leading segments in comparison to the UB approach, but lower outcomes for the segments in the end of a tonic pain event. However, with our approach, we are able to increase the performance on the segments which are not at the beginning or ending of a tonic event.

**Table 11:** Thermal domain: EFU ACL important features.

$f_{n}$	Feature name
$f_{1}$	Scl signal idr
$f_{2}$	Scl signal zero crossing
$f_{3}$	Scl signal split equal part std
$f_{4}$	Scl mean absolute value second diff
$f_{5}$	Scl 1. derivative signal sgm
$f_{6}$	Corr. 1. derivative signal zero crossing
$f_{7}$	Corr. 2. derivative signal zero crossing
$f_{8}$	Scl 1. derivative signal area min max
$f_{9}$	Scl 1. derivative signal mad
$f_{10}$	Corr. signal zero crossing

Further, as performed for the electric domain, we analyzed the ten most important features, with respect to the EFU signal. We applied the collection process as described in Section 7.1. The scores are depicted in Figure 3b. As it can be seen, for each approach, the feature-specific importance is different. Similar to the electric domain, we were able to create models which are shifted in direction of the tonic domain, which is observable by the changes in the scores and the good classification performance.

### 7.3 Pseudo-labeling in a clinical setting

In clinical settings, an APR system has to deal with various challenges, e.g. a new unknown hospitalized patient for which no labeled data is available, different types of pain such as acute or chronic pain or individual pain intensity levels. In such scenarios, it has to be assumed that the trained APR system is applied to completely unknown data, which may has a different data distribution. Therefore, the LOSO-CV testing protocol, as applied in our study, should be used for the evaluation of models which simulates a scenario of applying the classifier to new and unlabeled data of an unseen individual. In our study, we focused on the transfer task from phasic to tonic pain. Models trained on phasic pain domain data have to be adapted to the changing scenarios in a clinical setting since the body’s reaction might differ between phasic and tonic pain events (see Section 1). We propose the approach of using pseudo-labeling new unlabeled pain events, collected in a clinical setting, which are then incorporated into the training set to create an improved pseudo-labeling model. This leads to a classifier with an increased performance over time. Hence, with a pseudo-labeling approach, we are able to perform knowledge transfer from a generalized model to a more specialized classifier or, more generally, from one domain to another domain. For that specific task, we apply an additional processing step, which is the integration of classifier decisions over time into a more stable decision for tonic domain samples. This is a type of temporal classifier fusion that allows the recognition of pain based on varying observational lengths.

With an approach like ours, newly collected data without an assigned pain rating can be incorporated into the training set so that the classifier over time can be more and more transferred to the tonic pain domain. However, with the transfer task of phasic to tonic pain events, we are still in the beginning of this long-term research goal.

## 8 Conclusion and future work

In this study, we analyzed the classification performances in combination with various pseudo-labeling approaches, with respect to the adaptation of pain classifiers from phasic to tonic pain events. We evaluated the no pain vs. the highest pain intensity level task, specific to the electric and thermal domains. To this end, we applied a signal segmentation approach on the tonic domain samples, as performed in (22, 23). We achieved state-of-the-art results in combination with various signals whereby perfect pseudo labels might lead to reduced accuracy values. The best performing single modality in combination with the electric domain is the TRA signal (80.8%). For the thermal domain, the EFU modality performs best (70.0%). Moreover, we showed that outstanding results can be obtained for the pain duration adaptation task with hand-crafted features in combination with the Random Forest algorithm.

In addition, pseudo-labeling fusion approaches might increase performances as well as an adapted feature extraction for the EDA signal, as performed in (49, 81). Further, the evaluation of deep learning pseudo-labeling techniques have to be analyzed whereby the small amount of tonic domain samples has to be considered.

However, our findings indicate that, based on our settings, we are able to make the unlabeled tonic domain samples accessible for the training phase.

## Data Availability

The data analyzed in this study is subject to the following licenses/restrictions: The dataset is publicly available (for research applications) on request. Requests to access these datasets should be directed to sascha.gruss@uni-ulm.de.

## Footnotes

^1^The data set was recorded in compliance with the ethical guidelines settled in the World Medical Association Declaration of Helsinki (Ethical Committee Approval: 372/16) and approved by the ethics committee of the Ulm University, Germany.

^2^Based on the scikit-learn (80) function of the Random Forest implementation.
